# Age distribution of speech duration measures in healthy individuals with picture description tasks

**DOI:** 10.1002/alz70856_101085

**Published:** 2025-12-25

**Authors:** Junhyuk Louis Kwak, Mark Y Liberman, Sunghye Cho

**Affiliations:** ^1^ Linguistic Data Consortium, University of Pennsylvania, Philadelphia, PA, USA

## Abstract

**Background:**

Picture description tasks have been used to gauge communication capabilities of neurodegenerative patients. Previous studies have demonstrated that speech duration measures can help distinguish between healthy and neurodegenerative individuals, or even among patients of different neurodegenerative diseases. Hence, it is crucial to establish the baseline of those measures in healthy individuals in a wide age range, as deviations could indicate neurodegeneration. We collected picture description task responses from healthy volunteers to quantify such a baseline, which can aid early detection of neurodegenerative diseases.

**Methods:**

There were 290 healthy participants with a wide distribution of ages, from 15 to 90 years (M=49.9, SD=18.2), who voluntarily participated in picture description tasks online. The pictures included the Cookie Theft scene, the Picnic scene, and two similar pictures we designed; some participants completed all four, while others completed only some of them (M=2.6, SD=1.3). An in‐house speech activity detector program was employed to segment audio files into speech and pause segments automatically. We built linear mixed‐effects models to examine the effects of age on quantitative speech duration measures, including mean speech and pause segment durations, speech percentage (proportion of speech in the entire recording time), and pause rate (average pause count per minute). Sex, education levels, and picture types were included as fixed effects, and participant IDs as random effects.

**Results:**

Older participants showed lower speech percentages (β=‐0.079, SE=0.021, *p* <.001) and lower mean speech durations (β=‐0.004, SE=0.002, *p* = .022), but the decline failed to replicate in total speech duration (β=‐0.051, SE=0.079, *p* = .512). Meanwhile, pause rates increased with age (β=0.065, SE=0.022, *p* = .003), as with mean pause durations (β=0.002, SE=0.000, *p* = .001) and total pause durations (β=0.059, SE=0.023, *p* = .011). Total durations did not yield significant results (β=0.007, SE=0.095, *p* = .943).

**Conclusion:**

We demonstrated specific ageing trends across various speech duration measures. The different pictures and the demographic variance of participants support the reliability of our results. These outcomes contribute to the understanding of how speech duration patterns change with age, establishing a baseline to estimate the deviation from healthy ageing at different ages.

